# Inadequate Knowledge of Neonatal Danger Signs among Recently Delivered Women in Southwestern Rural Uganda: A Community Survey

**DOI:** 10.1371/journal.pone.0097253

**Published:** 2014-05-13

**Authors:** Jacob Sandberg, Karen Odberg Pettersson, Gustav Asp, Jerome Kabakyenga, Anette Agardh

**Affiliations:** 1 Social medicine and Global Health, Dept. of Clinical sciences, Lund University, Lund, Sweden; 2 Faculty of Medicine, Mbarara University of Science and Technology, Mbarara, Uganda; Seattle Childrens Hospital, United States of America

## Abstract

**Background:**

Early detection of neonatal illness is an important step towards improving newborn survival. Every year an estimated 3.07 million children die during their first month of life and about one-third of these deaths occur during the first 24 hours. Ninety-eight percent of all neonatal deaths occur in low- and middle-income countries like Uganda. Inadequate progress has been made globally to reduce the amount of neonatal deaths that would be required to meet Millennium Development Goal 4. Poor knowledge of newborn danger signs delays care seeking. The aim of this study was to explore the knowledge of key newborn danger signs among mothers in southwestern Uganda.

**Methods:**

Results from a community survey of 765 recently delivered women were analyzed using univariate and multivariate logistic regressions. Six key danger signs were identified, and spontaneous responses were categorized, tabulated, and analyzed.

**Results:**

Knowledge of at least one key danger sign was significantly associated with being birth prepared (adjusted OR 1.7, 95% CI 1.2–2.3). Birth preparedness consisted of saving money, identifying transportation, identifying a skilled birth attendant and buying a delivery kit or materials. Overall, respondents had a poor knowledge of key newborn danger signs: 58.2% could identify one and 14.8% could identify two. We found no association between women attending the recommended number of antenatal care visits and their knowledge of danger signs (adjusted OR 1.0, 95% CI 0.8–1.4), or between women using a skilled birth attendant at delivery and their knowledge of danger signs (adjusted OR 1.2, 95% CI 0.9–1.7).

**Conclusions:**

Our findings indicate the need to enhance education of mothers in antenatal care as well as those discharged from health facilities after delivery. Further promotion of birth preparedness is encouraged as part of the continuum of maternal care.

## Introduction

Early detection of neonatal illness is an important step towards improving newborn survival [1], [2]. Every year an estimated three million children die during their first month of life and about one third of these deaths occur during the first 24 hours. Ninety-nine percent of all neonatal deaths occur in low- and middle-income countries such as the east African nation Uganda, the location of this study. The leading causes are prematurity (35%), severe infections (27.5%), and asphyxia (22.5%) [3], [4]. A majority of these newborn deaths occur at home, indicating that few families recognize signs of newborn illness, and/or a majority of the neonates are not taken to health facilities when they are sick [5].

Neonates are more prone to show subtle signs of illness. Listlessness or difficulty feeding are sometimes the only signs present and illness may advance quickly [6], [7]. Different tools to facilitate identification of these health problems and reduce neonatal mortality have been introduced into health programs in several countries. Integrated Management of Newborn and Childhood Illness (IMNCI) developed by the World Health Organization (WHO) focuses on assessment of general danger signs in the examination of children presenting with illness at health care centers. The danger signs of severe illness included are 1) history of difficulty feeding, 2) movement only when stimulated, 3) temperature below 35.5°C, 4) temperature above 37.5°C, 5) respiratory rate over 60 breaths per minute, 6) severe chest in drawings and, 7) history of convulsions. Assessment of these signs will result in a high overall sensitivity and specificity for predicting the need for hospitalization of a newborn in the first week of life [8].

The use of the algorithm described above in community settings showed results when validated, suggesting that community screening by health workers should be encouraged [9]–[11]. Community-based postnatal health interventions such as home visits conducted by community health workers (CHW) have shown good results in studies conducted in Bangladesh, Pakistan, and India. They have resulted in reductions in newborn mortality rates of 30% to 61% [12]. While promising, scaling up this type of intervention in poor communities may have difficulties reaching satisfactory coverage, effectiveness and quality [13]. Interventions involving educational activities in women’s groups have been recommended for being low cost and easily scalable. A trained facilitator teaching 12 to 15 groups a month, (a total of 500 people) and recruiting newly pregnant women achieved reductions in newborn mortality of 30% to 32% and possibly a reduction in maternal mortality as well [14], [15]. A similar study conducted in Bangladesh using women’s groups showed no impact on newborn mortality, which might be due to a much lower coverage rate in the community (2%) [16]. The role of coverage magnitude is now being tested in a follow-up study [17]. A pooled analysis of all recent studies targeting women in community groups showed strong associations between these types of interventions, neonatal mortality rate (NMR), gains such as improved breastfeeding and care-seeking in other parts of essential newborn care [18].

Since much of this research is done in Asia, little is known about how to adapt the results to the cultural and regional contexts in sub-Saharan African (SSA) countries [19]. Studies elsewhere have shown that there is limited recognition of danger signs among mothers or other caregivers in low-income settings [1], [20], [21]. Poor knowledge is associated with delay in care-seeking, which is presented as the first delay in the three delays model, a model originally presented to explain the chain of events responsible for maternal deaths [22]. Using this model, Waiswa et al. estimated that delay in recognizing a problem and deciding to seek care accounted for half of all newborn deaths in eastern Uganda. The other delays contributing to newborn mortality are delay in reaching a health facility (Delay II) and delay in receiving adequate care (Delay III) [23].

Uganda has seen progress in reducing the under-five mortality rate (Millennium Development Goal 4) from 175 to 99 per 1000 between 1990 and 2011. However the reduction of neonatal mortality is not progressing adequately, the NMR is 26/1000 in Uganda, and many more newborns who survive infancy suffer life-long complications such as brain damage or lung problems [24].

In Uganda, 58% of all deliveries in 2011, were assisted by a skilled birth attendant (SBA). The Postnatal care coverage, however, is very low. Only 10.8% of Ugandan newborns receive any kind of health care within two days of birth, although antenatal care (ANC) is quite prevalent: 95% of all pregnant women have had at least one ANC visit among pregnant women [25]. Further, the evidence suggests that the newborn care in place is of poor quality. Studies have shown a serious neglect in essential newborn care practices such as cord care, thermal care, and feeding practices in eastern Uganda and other parts of SSA. Promoting breastfeeding, prevention of hypothermia, and community-based management of illnesses are all examples of interventions urgently needed at both the community and health facility level as part of universal coverage [26]–[28].

“Birth preparedness” is one of the strategies included in the *Roadmap for Accelerating the Reduction of Maternal and Newborn Mortality and Morbidity in Uganda* published by the Ugandan Ministry of Health [29]. It is aimed at empowering communities to increase their demand for health care. The term birth preparedness incorporates different preparations a woman can do during pregnancy in order to have the safest pregnancy and delivery possible. According to Johns Hopkins Program for International Education in Gynecology and Obstetrics (JHPIEGO), birth preparedness entails saving money, identifying a skilled provider or health facility for delivery and identifying transportation. Recognizing danger signs during pregnancy, childbirth, and infancy is also included [31]. The promotion and adoption of this strategy has been shown to increase the utilization of SBA at delivery, one of the important steps toward better maternal and newborn survival [30], [32].

To the best of our knowledge, no study has been undertaken to assess knowledge of neonatal danger signs and symptoms among women in the rural parts of Uganda. In addition, we know of no studies investigating the associations between birth preparedness and awareness of danger signs from a newborn health perspective. The overall aim of this study therefore was to a) determine the extent to which recently delivered women in southwestern Uganda could recognize newborn danger signs b) establish which factors or exposures predispose women to have a higher knowledge of newborn danger signs, and c) assess if birth preparedness is associated with a knowledge of newborn danger signs.

## Methods

### Setting

In 2009, 38% of Uganda’s population of 34.1 million was living below the poverty line of 1.25 USD/day [33]. The district of Mbarara covers 1788 square kilometers in the southwestern part of the country. The area is 80% rural and most of the 436 400 people who live there are engaged in peasant farming. The major city and only large urban area in the region is Mbarara. It contains a regional referral hospital linked to 46 health centers in the district. Data was also collected from two other administrative sectors in the district (Kashari and Rwampara counties), which are both mainly rural [34].

### Ethics Consideration

Permission was gotten from village leaders and prior to being interviewed, each participating woman signed or gave her thumbprint to a written consent form that was read to her. The Ugandan Council of Science and Technology and the Regional Ethical Review Board in Lund granted ethics clearance for the main project, proposed follow-up studies as well as the consent procedure.

### Sample and Data Collection

Data was collected between September 2010 and May 2011 as part of a community survey targeting women who were pregnant or had delivered within the last 12 months. The study was performed and planned as a pre-intervention study designed by JK, AA and KOP, partly in cooperation with Health Child Uganda. However no intervention has yet been done in this area. The data collection was performed by twelve research assistants with bachelor degrees in the social sciences. The assistants had been trained for one week on how to conduct interviews based on the safe motherhood questionnaire developed by the non-profit organization JHPIEGO [31]. Interviewees were randomly selected by means of two-stage cluster sampling. In the first stage, 120 villages were chosen and pregnant or recently delivered women within those villages were selected in the second stage. This was accomplished by identifying a starting point in the village and then choosing every other household until 10 women had been interviewed. In total 1199 pregnant and recently delivered women were interviewed. In order to assess the awareness of danger signs in newborns, only recently delivered women (765) were included in the study. The questionnaire was pretested in a neighboring district before data was collected in Kashari and Rwampara counties and only minor changes were introduced. The study utilized three of five sections of the questionnaire: socio-demographic information, reproductive history, and knowledge of pregnancy and childbirth. The survey questions and part of the database was also used in previously published studies [35]–[37].

### Variables

#### Background variables

*County of residence* was either “Kashari” or “Rwampara”. *Type of community* was coded as “rural” or “semi-urban”. *Level of education* was dichotomized as “less than secondary” and “ ≥secondary”. *Age* was dichotomized into “<25”and “≥25”. A low *household asset ownership* was applied when a household did not possess at least two of the following items: 1) radio, 2) television set, 3) mobile phone, and 4) bicycle, 5) motorcycle, 6) car/truck, and “moderate” when having ownership of at least two of the items. *Civil status* was dichotomized into “married” or “single”.

*Religion* was categorized as either “Christian” or “other”.

*Travel time to health facility* was coded “less than one hour” or “one hour or longer”.

#### Pregnancy- and delivery- related variables

*Recently delivered women* were women who had delivered in the previous 12 months.

*Use of skilled birth attendant* was coded “Yes” and “No”. “Yes” when the respondent stated that she had had assistance during delivery from either a midwife, a clinical officer or a doctor and “No” when answering that she was assisted by a “community health worker”, “traditional birth attendant”, “relative or friend” or “other” (including no one) [38].

The *number of ANC visits during pregnancy* was dichotomized. Women having attended the recommended number of ANC visits (four or more) were coded “≥4”, and those attending fewer visits were coded as “<4” [39], [40].

*Birth preparedness* was assessed by asking about the following preparations prior to delivery: 1) saving money, 2) identify transportation, 3) identifying skilled birth attendant, or 4) buying delivery kit or materials. Items one and two were calculated using women’s responses to the direct questions: “Did you or your family save money for the birth of this child?” and “Did you or your family identify transport for the birth of this child?” Question 3 included women who had either identified an SBA prior to delivery when asked “did you or your family identify a skilled provider for the birth of this child?” or had delivered with an SBA and had planned to do so. The fourth criterion included only women who reported that they had either bought a delivery kit or delivery materials. Those who said they had bought “clothes for the baby” or “food” were not categorized. To be classified as “well birth prepared”, at least three out of four preparations was needed.

### Women’s Knowledge of Newborn Danger Signs

Spontaneous responses to the question “In your opinion, what are some serious health problems that can occur during the first 7 days after birth that could endanger the life of a newborn baby?” were grouped and organized according to the definition presented by the WHO and the Young Infants Clinical Signs study group [41], [42]. These indicate an acute need for seeking professional health care. Seven groups of danger signs were identified: 1) difficulty feeding, 2) movement only when stimulated, 3) temperature below 35.5°C, 4) temperature above 37.5°C, 5) respiratory rate over 60 breaths per minute, 6) severe chest in drawings and 7) convulsions. We recoded “respiratory rate over 60 breaths per minute” and “severe chest in drawings” to “fast or difficulty breathing”. “Temperature above 37.5°C” was coded as “fever” and included the response “malaria”, since fever is the main symptom of that disease and is interpreted as a synonym to fever in this setting, as is also described from other African countries [25], [43].“Temperature below 35.5°C” was renamed “hypothermia” and included responses of “coldness”. The danger sign “movement only when stimulated” included responses “baby very tired”, “lethargic” and “unconscious”. Responses, which did not fit in any of these groups were not categorized. The number of “correct” signs mentioned by each woman was calculated and those who knew at least one of the key danger signs were grouped and further studied.

### Statistical Analysis

The data was coded and double entered into a database using Epi-Data Version 3.1. It was later transferred to SPSS version 21 for further cleaning and analysis. Individual responses were counted and displayed using frequency tables and cross tabulation. Associations between knowledge of at least one key newborn danger sign and socio-demographic and reproductive variables including birth preparedness were explored using univariate logistic regression. Crude odds ratios (OR) and 95% confidence intervals (CI) were presented. Multivariate logistic regression analysis was applied to investigate knowledge of danger signs in newborns and associated factors through stepwise adjustment for age, level of education, and household assets ownership. Birth preparedness was one of the main research questions and 1) attending ANC visits, 2) having high parity and 3) using an SBA were factors selected on basis of theoretical background. All factors were analyzed separately.

## Results

Data on 765 recently delivered women was entered into the study. Table 1 shows all the socio-demographic and reproductive variables. The participants were more commonly resident in a rural environment and were almost evenly divided between the two counties Kashari and Rwampara. Their mean age was 26.7 years, and 60.3% were 25 or older. Close to 23% had received secondary education and 27.6% came from households with “low” asset ownership. A majority, 72.4%, were categorized as having “moderate” asset ownership. A large majority of the interviewees were Christians (96.2%) and were currently married (95.0%). Sixty-seven percent of all women used an SBA during delivery. Table 2 displays, women’s knowledge of newborn danger signs. Knowledge of at least one of the defined key danger signs was present in 58.3% of all women: however, only 14.8% could name at least two signs. “Fast or difficulty breathing” was the most commonly known danger sign and referred to by almost 30% of the women. The response “fever” and “difficulty feeding” was given by approximately 20% of the women. The least known danger signs were “convulsions”, “movement only when stimulated” and “hypothermia”, stated by less than 5% of the respondents. Other responses were *ebino* (false teeth) and *Oburo* (millet disease) which is an explanation given to difficult breathing. Both of these often lead to various forms of traditional treatments such as surgical procedures to the gums or chest.

**Table 1:** Socio-demographic and reproductive characteristics of recently delivered Ugandan women (N = 765).

Characteristics	Number (n)	Percent (%)
County		
Kashari	389	50.8
Rwampara	376	49.2
Location of residence		
Rural	642	83.9
Semi-urban	123	16.1
Age (years)		
<20	56	7.3
20–24	247	32.3
25–29	226	29.5
≥30	125	16.3
≥35	111	14.5
Religion		
Christians	735	96.2
Other	29	3.8
Missing	(1)	
Marital status		
Married	727	95.0
Not married	38	5.0
Educational level		
<Secondary	589	77.1
≥Secondary	175	22.9
Missing	(1)	
Can read letter/Bible/newspaper		
Not at all	122	15.9
With difficulty	138	18.0
Easily	505	66.0
Household assets ownership		
Low (0–1 items*)	212	27.8
Moderate (≥2 items*)	553	72.4
Parity		
1	176	23.0
2–4	358	46.8
≥5	231	30.2
ANC Attendance		
<4 visits	234	31.1
≥4 visits	518	68.9
Missing	(13)	
Travel time to health facility		
<1 hour	419	55.3
≥1 hour	339	44.7
Missing	(7)	
Used SBA during delivery		
No	253	33.2
Yes	509	66.8
Missing	(3)	

*Radio, television set, mobile phone, bicycle, motorcycle and car/truck.

**Table 2:** Women’s knowledge of newborn danger signs (n = 765).

Danger sign	Number (n)	Percent (%)
Signs of severe illness (key danger signs)*		
Fast or difficulty breathing	225	29.4
Fever	159	20.8
Difficulty feeding	153	20.0
Convulsions	37	4.8
Movement only when stimulated	9	1.2
Hypothermia	2	0.3
*Knowledge of at least one key danger sign:*	446	58.3
*Knowledge of at least two key danger signs:*	113	14.8
Other:		
False teeth	86	11.3
Millet disease	24	3.1

*multiple responses.

The items included in the variable “birth prepared” are shown in Table 3. The most common way for women to prepare themselves for delivery was by saving money (87.8%), followed by identifying a skilled provider or health facility (64.3%) and identifying transport (60.1%). Only 20.7% of the women had bought childbirth material or a “mama-kit” prior to delivery. In total, 53.9% of the women had completed three out of four of the defined practices and were thereby considered to be “well birth prepared”.

**Table 3:** Recently delivered women’s reporting on birth preparedness in Uganda (N = 765).

	Number (n)	Percent (%)
Bought childbirth materials	158	20.7
Saved money	672	87.8
Identified transport	460	60.1
Identified skilled provider or health facility	492	64.3
Well birth prepared*	412	53.9

*Defined as having taken at least 3 of the 4 actions above.

As shown in the univariate logistic regression analysis in Table 4, no significant associations between knowing at least one danger sign and any socio-demographic characteristic were found. Further, no significant association was observed between knowledge of key danger signs and number of ANC visits (OR 1.0, 95% CI 0.8–1.4) or delivering with an SBA (OR 1.1, 95% CI 0.8–1.6). Being birth prepared was significantly associated with knowing at least one newborn danger sign (OR 1.6, 95% CI 1.2–2.1). The analysis concerning education, wealth, parity, use of ANC and use of skilled birth attendant was also done using three or four categories instead of two. However no major differences were found.

**Table 4:** Associations between socio–demographic, reproductive characteristics and knowledge of newborn danger signs.

Characteristics	Knowledge of at least 1key newborn danger sign n = 446/765 (%)	OR (95% CI)
County		
Kashari	231/389 (59.4)	1.0 (ref)
Rwampara	215/376 (57.2)	0.9 (0.7–1.2)
Location of residence		
Rural	378/642 (58.9)	1.0 (ref)
Semi–urban	68/123 (55.3)	0.9 (0.6–1.2)
Age		
<25	170/303 (56.1)	1.0 (ref)
≥25	276/462 (59.7)	1.2 (0.9–1.6)
Marital status		
Married	425/727 (58.5)	1.0 (ref)
Not married	21/38 (55.3)	0.9 (0.5–1.7)
Educational level		
<Secondary	349/589 (59.2)	1.0 (ref)
≥Secondary	96/175 (57.0)	0.8 (0.6–1.2)
Missing	(1)	
Household assets ownership		
Low (0–1 items)	126/212 (59.4)	1.0 (ref)
Moderate (≥2 items)	320/553 (57.9)	0.9 (0.7–1.3)
Parity		
1–2	175/318 (55.0)	1.0 (ref)
≥3	271/447 (60.6)	1.2 (0.9–1.7)
*Travel time to health facility*		
<1 hour	243/418 (58.1)	1.0 (ref)
≥1 hour	200/339 (59.0)	1.0 (0.8–1.4)
Missing	(7)	
ANC attendance		
<4 visits	135/234 (57.7)	1.0 (ref)
≥4 visits	304/518 (58.7)	1.0 (0.8–1.4)
Missing	(13)	
Used SBA during delivery		
No	142/253 (56.1)	1.0
Yes	302/509 (59.3)	1.1 (0.8–1.5)
Missing	(3)	
Birth prepared		
No	184/353 (52.1)	1.0 (ref)
Yes	262/412 (63.6)	1.6 (1.2–2.1)

In Table 5, adjustments for the background variables age, education and ownership of household assets were performed using multivariate logistic regression analysis. The adjusted OR for having high parity (OR 1.2, 95% CI 0.8–1.7), ANC attendance (OR 1.0, 95% CI 0.8–1.4) and delivering with an SBA (OR 1.2, 95% CI 0.9–1.6) only slightly differed from the crude OR.

**Table 5:** Multivariate logistic regression on knowledge of danger signs and separate independent factors.

Factors of special interest	Model 1:OR(95% CI)(adjusted for age)OR (95% CI)	Model 2: OR(95% CI)(adjusted for ageand education)	Model 3: OR(95% CI)(adjusted forage, education and householdassets ownership)
Birth prepared	1.6 (1.2**–**2.2)	1.6 (1.2**–**2.2)	1.7 (1.2**–**2.2)
Parity	1.2 (0.9**–**1.8)	1.2 (0.8**–**1.7)	1.2 (0.8**–**1.7)
ANC visits	1.0 (0.8**–**1.4)	1.0 (0.8**–**1.4)	1.0 (0.8**–**1.4)
Used SBA	1.2 (0.9**–**1.6)	1.2 (0.9**–**1.6)	1.2 (0.9**–**1.6)

The association between knowledge of newborn danger signs and birth preparedness remained statistically significant when the three socio-demographic factors were introduced into the model (OR 1.7, 95% CI 1.2–2.2). Moreover, after adjusting for high parity, ANC attendance and delivering with an SBA the significant association persisted (data not shown).

## Discussion

The main findings of this study was that recently delivered women in Uganda had poor knowledge of key newborn danger signs, whereas knowing at least one of those signs was significantly associated with being well prepared for birth. Surprisingly, increased knowledge of newborn danger signs was not detected neither among women attending the recommended number of ANC visits nor among women using an SBA at delivery.

Agarwal et al. (2010) who also reported that birth preparedness was significantly associated with knowledge of at least one key newborn danger sign similarly found a significant association between birth preparedness and knowledge of danger signs (OR 1.8, 95% CI 1.1–3.2). However, the significance did not remain when adjusting for other independent variables [44]. In our study, the association appeared to be unaffected by any confounders. Possible explanations for this difference could be other definitions of birth preparedness and danger signs as well as a smaller sample size in Agarwal’s study. However, since the author does not present the adjustment stepwise, it is difficult to identify the impact of individual confounders.

Previous studies concerning women’s level of knowledge of neonatal danger signs have shown differences between, as well as within, settings. In a study conducted in Pakistan knowledge of the danger signs “difficulty feeding” and “difficulty breathing” correlated with the findings of our study. However, other findings from the same study such as knowledge of “convulsions”, “hypothermia” and “fever” indicate a higher overall knowledge in the Pakistani community than in our population [45].

An Indian study by Dongre et al. (2009) found slightly greater awareness than our study with regard to knowledge of “difficulty feeding” (22.2% versus 20.0%), “convulsions” (9.7% versus 5.0%) and “hypothermia” (2.5% versus 0.3%). However, about 75% of the respondents in India named “fever” as a danger sign, which is considerably higher than in our population [46]. Awasthi et al. (2006) also reported high knowledge of fever, (approximately 90%) from another part of India [20]. The variations might be explained by differences in the disease spectrum between Uganda and India, a varying focus in the health education provided to mothers or community interventions aimed at caregivers. For example, there seems to be more awareness of “fever” as a danger sign in Indian communities, as reported above. Even though the most common cause of fever in the newborn is neonatal sepsis [8], greater awareness of fever in Uganda, a country with high malaria prevalence, might have been expected. The infrequent mention of fever found in our study may be due to the fact that malaria and subsequently fever is not considered to be a serious condition by the communities. This has previously been documented in studies from other SSA countries as well as in one study conducted in the Mbarara region [47]–[50]. In summary, our results are in line with findings from other low-income settings, even though differences are present. The poor knowledge of newborn danger signs we recorded may be an important cause of delay in care seeking (Delay I) in agreement with Waiswa et al., who argue that Delay I is the underlying cause for up to 50% of neonatal deaths in eastern Uganda [23]. However, as stated by Hill et al., [2] symptom recognition might not always be necessary for care seeking and even when danger signs are recognized, it might not result in seeking appropriate care. A danger sign could, for example, be misinterpreted, leading to treatments with traditional methods. Moreover, the decision to seek hospital care might be made by family members, such as grandmothers or husbands.

The lack of association between higher education and increased knowledge of newborn danger signs in our study was unexpected. No previous studies of this particular association were identified. A previous study found that knowledge of obstetric danger signs was significantly higher among educated women [51]. An explanation for our results might be that the overall understanding of newborn health in the community is very low, which does not give more educated mothers much of an advantage.

In our study a higher proportion of women delivered with the assistance of an SBA than the national mean (66% versus 53%) presented in the Ugandan Demographic Health Survey for the rural parts of the country. However the figure was lower than the urban population where 89% of women deliver using an SBA [25]. The higher number might indicate a positive development in favor of skilled birth attendance in the rural Mbarara region compared to the rest of the country. The univariate and multivariate analysis showed a small but not significant improvement in knowledge of newborn danger signs following exposure to an SBA. Deciding to deliver with an SBA is a decision expected to return multiple health benefits. Information about newborn danger signs and the circumstances that necessitate newborns returning to the health care unit should be passed over to the mothers at discharge. This is an opportunity that should not be missed [52]. In view of our findings, the extent and quality of the information given to women at present with regard to their newborn in relation to the newborn’s health and what danger signs to look for needs significant improvement.

Despite a high ANC attendance among study participants, our findings showed no increased knowledge of newborn danger signs after four visits or more. This gives rise to great concern, as previous studies conducted in Laos and Malawi indicate that by providing structured education during the ANC period, knowledge of events and danger signs in all phases of pregnancy and the postnatal period improves [53], [54]. However, recent studies on antenatal care in Uganda have reported on ineffective organization of educational sessions and poor counseling for both birth preparedness and risk factors [55], [56]. Our study adds further concerns about the quality of ANC in Uganda.

We found that almost 9 out of 10 women in the Mbarara district had saved money in preparation for delivery, which is higher than the percentage in India (76.9%) and Burkina Faso (83.3%). With regard to identifying an SBA or health facility our population (64.3%) was just below India (69.6%) and far above Burkina Faso (43.9%). More women had identified transport in our study (60.1%) than in the other settings [32], [44]. Saving money in anticipation of delivery seems to be a widespread practice in the Ugandan communities included in this study but buying a birth kit or delivery materials was surprisingly low, raising questions about the availability of birth kits in the community.

The false teeth (11%) and millet disease (3%) responses some women gave have both previously been described in studies as common myths leading to contacts with traditional healers and the use of potentially life threatening practices to the newborn, such as extraction of “teeth” and cuttings. Our findings indicate that these perceptions are still present in the community and should be addressed and opposed by education [57], [58].

### Strengths and Limitations of Study

The relatively high number of recently delivered women used in this study and the consistent use of research assistants with extensive local knowledge are part of this study’s strengths. There are, however, some limitations.

Since this study used unprompted spontaneous answers it may not reflect women’s recognition of the danger signs we defined if they were to appear in the newborn. Recognition of danger signs may be higher with another study design, such as using a list of danger signs or images and having the respondent point out which ones might endanger the life of the newborn. Also by using prompted answers, i.e., direct questions about each danger sign; the knowledge shown is expected to be higher.

There is the further possibility that some women did not understand the questions fully, or that difficulties arose in translation. However, research assistants were alerted to note the exact answer when dealing with medical terms or words in the local language that would be hard to translate correctly. A professional translator with medical experience was hired during data cleansing in order to correctly translate the answers thus minimize the risk of information loss during translation.

The findings have all been self-reported, which may be problematic in the case of recall error. It is also possible that the subgroup of women who had delivered within one month of the survey had a better recollection than those who delivered up to one year earlier. Had we chosen women had delivered more recently it may have been possible to minimize this type of error.

The use of SBA in this study considers only the person engaged to perform this service, and not the delivery location, which also is of considerable significance. However, since this variable is based solely on the statements of the women interviewed, with no possibility of judging whether the delivery environment or competence of the health care professionals present was suitable, it is only the best estimate available. This is a problem widely debated in the research community and a good definition and measurement is currently lacking, and it adds a degree of uncertainty to our results [30], [59].

## Conclusion

The findings of our study show poor understanding of danger signs in the studied area. This indicate a need to enhance educational efforts aimed at all pregnant and delivered women in the community. Promotion of birth preparedness is also encouraged as it has been shown to alert women to newborn danger signs. Studies using intervention methods could further establish the connection with knowledge of a danger signs and newborn survival as well as the role of other family members in identifying newborn illness. Finally, women’s groups have been effective educational vehicles according to studies elsewhere and could also be used in Uganda in order to heighten awareness of newborn healthcare in the communities.
